# Fabrication and Characterization of Immature Porcine Cartilage-Derived Cell Biomembranes

**DOI:** 10.3390/jfb16030092

**Published:** 2025-03-05

**Authors:** Phuong-Vy Bui, Vang Pham Thi, Trung-Nhan Vo, Viet-Trinh Nguyen, Thai-Duong Tran, Vy-Khanh Vo, Phuong Le Thi, Dieu Linh Tran, Minh-Dung Truong

**Affiliations:** 1 Biotechnology Center of Ho Chi Minh City, Ho Chi Minh City 700000, Vietnam; buiphuongvy2002@gmail.com (P.-V.B.); ptvang.snn@tphcm.gov.vn (V.P.T.); votrungnhan17@gmail.com (T.-N.V.); ntviettrinh2001@gmail.com (V.-T.N.); tranthaiduong0510@gmail.com (T.-D.T.); vovykhanh041200@gmail.com (V.-K.V.); 2 Institute of Applied Materials Science, Vietnam Academy of Science and Technology, Ho Chi Minh City 700000, Vietnam; ltphuong@iams.vast.vn; 3 Institute of Chemical Technology, Vietnam Academy of Science and Technology, 1A TL29 Street, Thanh Loc Ward, District 12, Ho Chi Minh City 700000, Vietnam; tdlinh92@gmail.com; 4 Faculty of Applied Sciences, Ton Duc Thang University, Ho Chi Minh City 700000, Vietnam

**Keywords:** autologous chondrocyte implantation, biomembrane, one-day-old porcine cartilage

## Abstract

(1) Background: Knee cartilage injury is at the top of the rising concerns among bone and joint disorder patients. Autologous chondrocyte implantation (ACI) is widely used to approach knee cartilage deterioration. Integrating autologous chondrocytes and periosteal patches aids in forming new cartilage-like tissue at the lesion area. This study uses a novel cell source from one-day-old porcine cartilage to fabricate a biomembrane as a substitute for periosteal membranes in cell implantation techniques for treating knee cartilage injuries. (2) Methods: Cells isolated from one-day-old porcine cartilage tissue were identified and assessed for their proliferation capability, differentiation ability, and membrane formation potential. The protein component of the biomembrane was also defined by proteomics. The cartilage repair ability was also confirmed using an in vitro transplantation model. (3) Results: Negative results for porcine infectious diseases are pivotal in selecting suitable piglets to provide cartilage tissue. The cells successfully obtained from one-day-old porcine cartilage exhibited stem-cell-like characteristics (CD34-, CD45-, CD90+, CD105+), including a high proliferation to 20 passages (doubling time: 1–2 days) and a capacity to differentiate into various cell types (osteogenesis, adipogenesis, and chondrogenesis). The stem cells were successfully applied in the fabrication of the biomembranes. The protein components of the biomembrane included an extracellular matrix and growth factors. The in vitro transplantation model showed that the biomembrane induced the repair ability of cartilage defects. (4) Conclusions: This study is the first to successfully harvest stem cells from one-day-old porcine cartilage for biomembrane fabrication for a knee cartilage injury therapeutic application.

## 1. Introduction

Knee osteoarthritis (OA) is a prevalent condition globally. Osteoarthritis is a progressive condition that deteriorates the knee’s cartilage, resulting in discomfort, a limited range of motion, and inflexibility [1]. The onset of knee osteoarthritis can be influenced by various factors, including aging, obesity, previous joint injuries, and genetic predisposition [2]. Therefore, optimizing treatment methods for OA has become an essential and urgent demand in recent decades.

Indeed, numerous interventions have been researched and clinically applied to enhance function and restore activity at the injury site for patients with OA. These include non-pharmacological treatments (physical therapy regimens) [3], pharmacological approaches (utilizing medications, cytokines, etc.) [4,5,6,7,8,9], and surgical interventions (involving different surgical techniques) [10,11,12,13,14]. The treatment methods for patients with OA are tailored based on the extent and severity of the injury. Autologous cell implantation (ACI) has emerged as a promising approach for treating OA. This method uses the patient’s cells to promote tissue regeneration and repair. The success of this method relies on several factors, with the most critical being the source of autologous cells and the use of periosteal patches to facilitate the formation of regenerative tissue with hyaline cartilage-like characteristics. The type and the quality of the cells used are crucial. Frequent sources of mesenchymal stem cells (MSCs) encompass bone marrow, adipose tissue, and peripheral blood. These cells have the potential to differentiate into cartilage cells and support tissue regeneration [8,11,12]. These patches are used to create a supportive environment for the implanted cells. Periosteal patches contain progenitor cells and growth factors that facilitate the formation of regenerative tissue with hyaline cartilage-like characteristics. They provide a scaffold for cell attachment, proliferation, and differentiation, enhancing the overall effectiveness of the treatment. Research on improving periosteal patches or substituting them with biomembranes is still in its early stages and remains limited. While periosteal patches have shown promise in enhancing tissue regeneration, ongoing efforts exist to develop and optimize biomembranes as potential substitutes [15].

Studies have explored using biomembranes that replicate the periosteum’s structural and functional properties, aiming to harness the body’s natural healing processes [16]. These biomembranes are designed to provide a supportive environment for cell growth and tissue regeneration, similar to periosteal patches. However, more research is needed to fully understand their efficacy and long-term outcomes [17].

The decellularization of source tissues is necessary for preparing biological scaffold materials from mammalian ECM, usually utilizing a combination of detergents, enzymes, and physical forces. Maximal decellularization is necessary as the source tissues are usually of allogeneic or xenogeneic origin. The host tissue response following implantation depends on the efficacy of decellularization and the removal of cell remnants. Establishing standards based on the quantitative criteria of the remaining nuclear material is reasonable to assess the efficacy of decellularization. Biologic scaffolds have the potential to provide benefits in tissue engineering and regenerative medicine if optimal decellularization methods are employed [18].

On the other hand, stem cells isolated from immature tissue (immature tissue-derived cells) are known as a safe cell source, similar to autologous cells in grafting. These cells exhibit superior proliferation and differentiation capabilities compared to adult stem cells. The function and characteristics of adult stem cells can be unstable due to their isolation from various donors; however, stem cells isolated from immature tissue can proliferate into large quantities of functionally and characteristically similar cells due to a single donor origin. Furthermore, stem cells derived from immature tissue exhibit embryonic-like functionality while maintaining a safety profile comparable to adult stem cells [19,20,21]. Owing to these attributes, stem cells from immature tissue demonstrate a high potential for application in cell therapy.

This study used a novel cell source from one-day-old porcine cartilage to fabricate a biomembrane to alter periosteal membranes in cell implantation techniques for treating knee joint injuries. The research involved isolating and characterizing cells from one-day-old porcine cartilage. These cells’ proliferation and differentiation capacities and their ability to form biomembranes were also assessed. The protein component of the biomembrane also was defined by proteomics. The cartilage repair ability was also confirmed using an in vitro transplantation model.

## 2. Materials and Methods

### 2.1. Collection and Isolation of Stem Cells from Cartilage Tissue of One-Day-Old Pig

This study obtained healthy one-day-old pig with intact limbs from a local certified farm that adheres to strict animal welfare and health regulations. The knee joints of the pig were obtained immediately after sterilization and surgery. The newly obtained cartilage tissue was rinsed three times with 10 mL of a 1× phosphate-buffered saline (PBS) solution. (Gibco, Waltham, MA, USA). The cartilage tissue was then finely chopped into pieces smaller than 1 mm. A collagenase solution (0.1% collagenase, Worthington, OH, USA) was added and placed in a cell culture incubator (conditions: 37 °C, 5% CO_2_) for 16 h to degrade the fibrous structures in the tissue and liberate single cells. After the 16 h incubation, the digested tissue was centrifuged to collect the single cells accumulating at the tube bottom, which were then washed three times with a 1× PBS solution. The separated single cells were subsequently resuspended in a vital culture medium consisting of Dulbecco’s Modified Eagle Medium (DMEM) supplemented with 10% fetal bovine serum (FBS) and 1% Penicillin–Streptomycin (Gibco, Waltham, MA, USA).

Single cells derived from cartilage tissue were counted using trypan blue solution (Sigma, Waltham, MA, USA) and a hemocytometer to distinguish and count the live (unstained) and dead (blue-stained) cells under a microscope (Nikon, Tokyo, Japan). Then, the cells were cultured in a suitable culture medium in a cell culture incubator. The cell morphology was observed through an inverted microscope, and the culture medium was refreshed every three days during the cultivation period.

### 2.2. The Evaluation of the Biological Characteristics of the Cells Collected from One-Day-Old Porcine Cartilage Tissue

Using an inverted microscope, the cells isolated from the one-day-old porcine cartilage tissue were observed for morphology across passages (up to passage 20). The proliferation rate of the cells was assessed by calculating their doubling time at each passage, up to passage 20. The cells were harvested and counted regularly using a hemocytometer and the trypan blue exclusion method to distinguish live cells from dead ones. The doubling time of a cell population can be calculated using the following formula:

Doubling time (*Td*) = (*t* × ln(2))/(ln(*Nt*/(*No*)))

*Td* is the doubling time.

*t* is the period during which the cells are growing.

*No* is the initial number of cells.

*Nt* is the number of cells at time t.

Cell identification was performed by expressing cell surface markers evaluated by FACS, including CD34, CD45, CD90, and CD105 (Thermo Fisher, Morecambe, UK).

### 2.3. The Evaluation of the Differentiation Potential of the Stem Cells from One-Day-Old Porcine Cartilage Tissue

The cells at passage 2 (P2) were evaluated for their ability to differentiate into three basic cell lineages: adipogenesis, osteogenesis, and chondrogenesis. The cells were cultivated in an adipogenic differentiation medium for adipocyte differentiation, with their adipogenic potential being evaluated through Oil Red O staining. Additionally, the cells were grown in an osteogenic differentiation medium for osteocyte differentiation, and their osteogenic potential was assessed using Alizarin Red staining. For chondrocyte differentiation, the cells were cultured in 3D structural spheroids using a chondrogenic differentiation medium, and their chondrogenic potential was assessed using Safranin-O staining.

### 2.4. The Evaluation of the Biomembrane-Forming Capability of the Stem Cells from One-Day-Old Porcine Cartilage Tissue

The cells at passage 2 (P2) were grown in 6-well plates at a density of 0.5 × 10^6^ cells/cm^2^. The membrane formation medium consisted of a cell culture medium supplemented with insulin–transferrin–selenium (ITS), 50 mg/mL ascorbate 2-phosphate, 100 nM dexamethasone, 40 mg/mL proline, and 1.25 mg/mL BSA (Sigma, Waltham, MA, USA). After two weeks of culturing, the cell sheets were decellularized using a 1% SDS solution (Sigma, Waltham, MA, USA). Genetic material was eliminated using DNAse/RNAse enzymes. The DNase (Thermo Fisher Scientific, Waltham, MA, USA) concentration was 50 U/mL, and the RNase (Sigma, Waltham, MA, USA) concentration was 100 µg/mL. The cell sheets were freeze-dried at −80 °C to create the PCM biomembrane.

### 2.5. Proteomic Analysis in PCM Biomembrane

The biomembrane underwent lysis to release its protein content. The extracted proteins were digested with trypsin to generate peptides. These peptides were subsequently analyzed using matrix-assisted laser desorption ionization time-of-flight mass spectrometry (MALDI-TOF MS, Billerica, MA, USA) to identify and quantify the present proteins. The generated mass spectrometry data were used to establish a genetic database. This database was subsequently analyzed using the Gene Ontology enrichment analysis and visualization tool (GOrilla) to determine the biological processes, cellular components, and molecular functions associated with the identified proteins.

### 2.6. The Evaluation of the Cartilage Repair Capacity of the PCM Biomembrane

The cartilage repair capacity of the biomembrane was evaluated using an in vitro transplantation model. A 3 mm cartilage defect was drilled into a porcine cartilage block with a diameter of 6 mm. The experiment included four groups: (i) the untreated group: a control group with no treatment (*n* = 5); (ii) the cells-treated group: chondrocytes were used to treat the cartilage defect (*n* = 5); (iii) the PCM-treated group: the PCM biomembrane was used to treat the cartilage defect (*n* = 5); and (iv) the cells/PCM-treated group: chondrocytes and the PCM biomembrane were used to treat the cartilage defect (*n* = 5). The porcine cartilage blocks were cultured in a chondrogenic medium and maintained in a cell culture incubator under 37 °C and at 5% CO_2_ for three weeks. The culture medium was refreshed every three days during cultivation. The cartilage repair was assessed three weeks after transplantation using Safranin-O staining to evaluate the presence and quality of repaired cartilage tissue.

### 2.7. Statistical Analysis

Data were expressed as the mean ± standard deviation. The graphic images were created and the statistical analyses were conducted using GraphPad Prism 8 version 8.0.2 (GraphPad, San Diego, CA, USA) with a one-way analysis of variance, applying Tukey’s multiple comparisons or unpaired *t*-tests.

## 3. Results

### 3.1. Collection and Isolation of Stem Cells from Cartilage Tissue of One-Day-Old Pig

Cartilage tissue was collected from the knee joints of a one-day-old pig, with a cartilage tissue mass of 0.90 ± 0.27 g. The number of isolated cells was 28 ± 8.51 million, the number of isolated cells per gram of cartilage tissue was 31.03 ± 4.39 million, and the number of cells obtained at P0 was 100 ± 30.41 million. The cell morphology after isolation was consistent across the samples, as shown in Supplementary Figure S1. The cells at P0 were collected and stored in liquid nitrogen.

### 3.2. The Evaluation of the Biological Characteristics of the Cells Collected from One-Day-Old Porcine Cartilage Tissue

**Figure 1:**
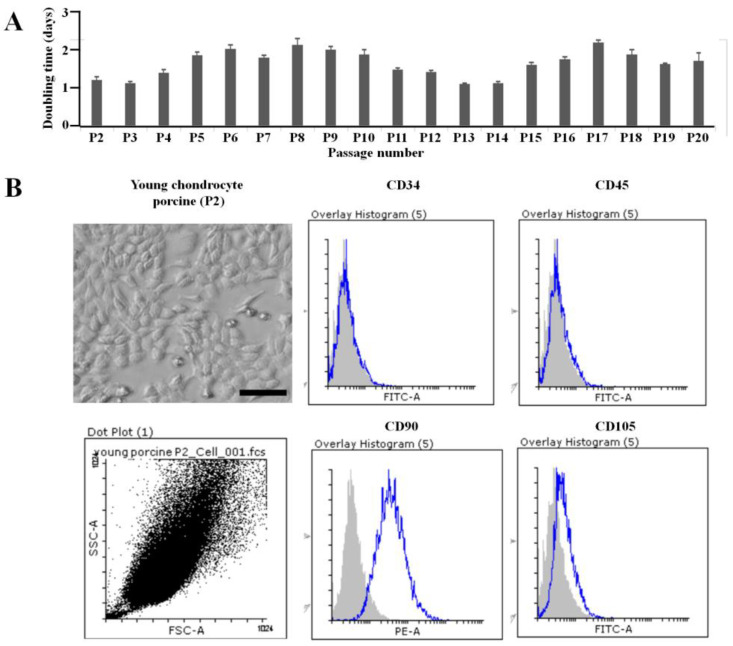
The biological characteristics of cells collected from one-day-old porcine cartilage tissue. (**A**) The cell proliferation rate, measured by the doubling time, ranged from 1 to 2 days and was observed from stage P1 to P20. (**B**) At passage 2 (P2), the cells expressed the following surface markers: CD34 (0.19 ± 0.18%); CD45 (0.28 ± 0.19%); CD90 (95.57 ± 0.48%); and CD105 (9.58 ± 1.48%) (scale bar: 100 µm).

The cells were thawed and monitored for morphology under a microscope until the 20th passage (P20) (Supplementary Figure S1). The shape and size of the cells from stages P1 to P20 did not show significant changes. The cell proliferation rate, measured by the doubling time, ranged from 1 to 2 days and was observed from stage P1 to P20 (Figure 1A). Therefore, the morphological characteristics and proliferation capacity of the cells from the cartilage tissue of the one-day-old pig remained stable throughout stages P1 to P20.

The cells were characterized using flow cytometry, as illustrated in Figure 1B. At passage 2 (P2), they displayed the following surface markers: CD34 (0.19 ± 0.18%), CD45 (0.28 ± 0.19%), CD90 (95.57 ± 0.48%), and CD105 (9.58 ± 1.48%).

### 3.3. The Evaluation of the Differentiation Potential of the Stem Cells from One-Day-Old Porcine Cartilage Tissue

**Figure 2:**
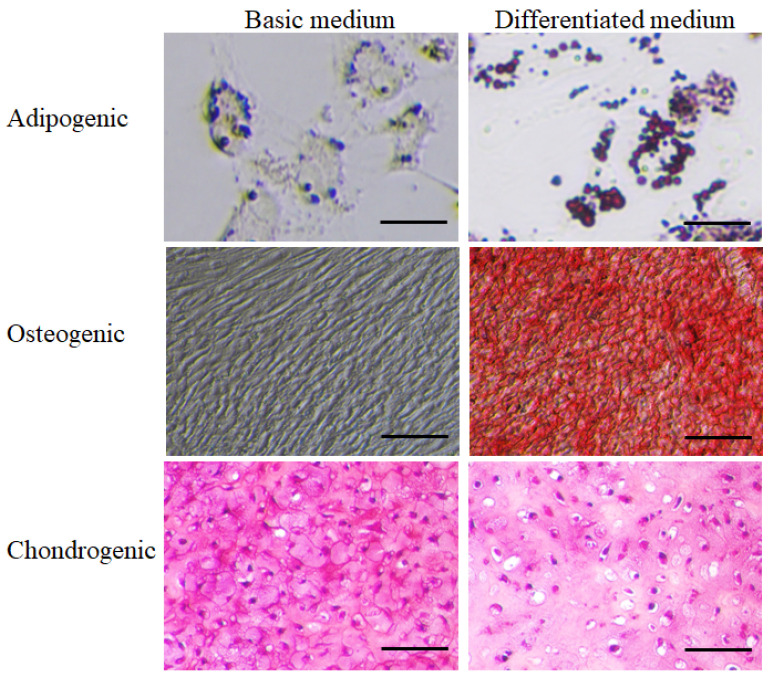
The differentiation potential of stem cells from one-day-old porcine cartilage tissue. Adipogenic differentiation: The ability to form adipocytes was observed using Oil Red O staining in a differentiation medium. Red-stained lipid droplets were observed after 14 days of culturing in the differentiation medium, compared to the negative control, where they were not observed (scale bar: 10 µm). Osteogenic differentiation: The ability to form osteocytes was observed using Alizarin Red staining in an osteogenic differentiation medium. The red staining of Alizarin Red confirmed differentiation into osteocytes observed after 21 days of culturing in the differentiation medium, compared to the negative control, where staining was not observed (scale bar: 50 µm). Chondrogenic differentiation: Thin sections of the 3D tissue samples showed staining with Safranin-O in both media (scale bar: 50 µm).

Adipogenic differentiation: The ability to form adipocytes was observed using Oil Red O staining in a differentiation medium. Red-stained lipid droplets were observed after 14 days of culturing in the differentiation medium, compared to the negative control, where they were not observed (cells not differentiated into adipocytes in the basic culture medium) (Figure 2).

Osteogenic differentiation: The ability to form osteocytes was observed using Alizarin Red staining in an osteogenic differentiation medium. The red staining of Alizarin Red confirmed differentiation into osteocytes, which was observed after 21 days of culturing in the differentiation medium, compared to the negative control, where staining was not observed (cells not differentiated into osteocytes in the basic culture medium) (Figure 2).

Chondrogenic differentiation: Cells were successfully cultured into 3D aggregates in the basic culture medium and the chondrogenic differentiation medium. In both media, thin sections of the 3D tissue samples showed staining with Safranin-O. However, chondrocytes and lacunae (small cavities containing chondrocytes) were more abundantly formed in the chondrogenic differentiation medium. In contrast, lacunae were not formed in the basic culture medium (Figure 2).

### 3.4. The Evaluation of the Biomembrane-Forming Capability of Stem Cells from One-Day-Old Porcine Cartilage Tissue

**Figure 3:**
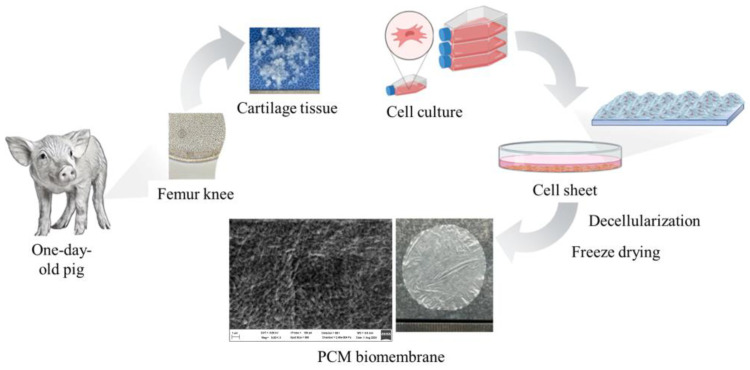
The biomembrane-forming capability of the stem cells from one-day-old porcine cartilage tissue. The cells derived from one-day-old porcine femur knee cartilage tissue formed the cell sheet. The PCM biomembrane, harvested after decellularization and freeze-drying processes, showed the fiber structure in the SEM image.

The cells were successfully formed by the P2 sheets at a density of 0.5 × 10^6^ cells/cm^2^ in the supplemented cell culture medium. After two weeks of culturing, the cell sheets were decellularized using a 1% SDS solution. The decellularization process effectively removed cellular components, as confirmed by the absence of cellular debris under microscopic examination. Genetic material was eliminated using DNAse/RNAse enzymes, ensuring the removal of residual DNA and RNA. The decellularized cell sheets were freeze-dried at −80 °C to create the PCM biomembrane. The resulting biomembrane exhibited a uniform structure and retained its mechanical integrity, making it suitable for further applications in tissue engineering and regenerative medicine (Figure 3).

### 3.5. Proteomic Analysis of PCM Biomembrane

The results indicated the expression of specific proteins in the PCM biomembrane. The proteomic profile allows for identifying and analyzing these proteins, providing insights into their response to various treatments. This information is essential for understanding the underlying biological processes and informing future experimental designs and therapeutic strategies. Extracellular matrix protein 1 (ECM1) was the most prominent among the identified proteins. Additionally, several growth factors were identified, including transforming growth factor (TGF), fibroblast growth factor (FGF), insulin-like growth factor (IGF), and epidermal growth factor (EGF) (Table 1).

### 3.6. The Evaluation of the Cartilage Repair Capacity of PCM Biomembrane

**Figure 4:**
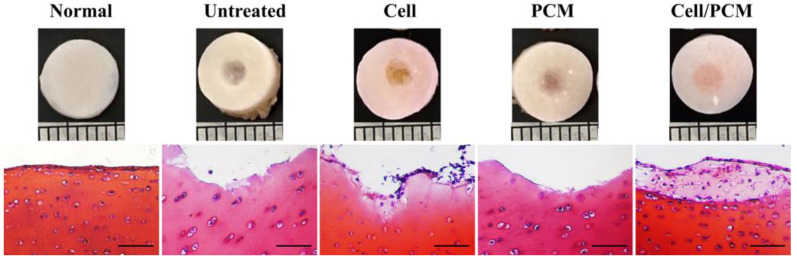
The cartilage repair capacity of the PCM biomembrane. Cross-imaging and Safranin-O staining revealed the formation of new cartilage tissue in the defect area. No new tissue was observed in the defect area in the untreated group. In the cells-treated group, new tissue with a small number of cells was present in the defect area. No new tissue was observed in the defect area in the PCM-treated group. In the cells/PCM-treated group, new tissue formed in the defect area, with the cells resembling chondrocytes and the lacunae stained with Safranin-O (scale bar: 50 µm).

Cross images and Safranin-O staining showed the formation of new cartilage tissue in the defect area. No new tissue was observed in the defect area in the untreated group, and the damaged surface was uneven. This group served as a control group to understand the impact of not treating the defect. New tissue with a few cells appeared in the defect area in the cell-treated group, and the damaged surface was uneven. This result indicated the limitation of healing the defect if the treatment lacks cell stabilization at the injury site. In the PCM-treated group, no new tissue formed at the defect; however, the cartilage surface at the injury site was smooth, indicating the PCM treatment’s efficacy in limiting damage. The structural uniformity, with notable differences compared to the normal sample, suggests the impact of the PCM treatment. In the cell/PCM-treated group, the combination of cells and the PCM indicated a synergistic or combined treatment effect. New tissue formed in the defect area, with the cells resembling chondrocytes and the lacunae stained with Safranin-O (Figure 4).

## 4. Discussion

This study demonstrated a novel approach using cells derived from one-day-old porcine cartilage to fabricate a biomembrane for inducing cartilage repair in knee joints. The cells were successfully harvested from a healthy one-day-old pig and exhibited high proliferation and renewal, as well as multiple differentiation capabilities. These characteristics are akin to stem cells, essential for forming biomembranes. The protein profile analysis further revealed the presence of extracellular structure organization, extracellular matrix organization, collagen fibril organization, and supramolecular fiber organization. Additionally, the PCM biomembrane showed promising results in inducing cartilage repair in joint damage, highlighting its potential as an effective treatment for knee joint injuries.

One-day-old porcine cartilage was utilized in this research due to its superior cell yield efficiency, proliferation capacity, and differentiation ability. The results demonstrated that collecting and isolating cells from the cartilage tissue of a one-day-old pig is highly stable, as evidenced by the high number of isolated cells per gram of cartilage tissue and the high yield of cells, which increased well until passage 20. These cells also exhibited stem cell marker expression and multipotent differentiation into chondrogenic, osteogenic, and adipogenic lineages.

Research indicates that immature cartilage progenitor cells have been explored as a viable cell source for applications in regenerative medicine [20,21]. Cartilage cells are harvested from different developmental stages, with varying results regarding differentiation abilities [22,23]. Mijin Kim et al. demonstrated that the characteristics and abilities of immature cartilage cells depend highly on their developmental stages, which should be considered when developing fetal cell-based therapies. The expression of pluripotency genes (Nanog, Oct4, and Sox2) was detected on embryonic day 14 in the rat model, while the expression of chondrogenic genes (Col2a1, Acan) progressively increased in cartilage cells until the formation of epiphyseal cartilage on day 20 in the rat model. This implies that cartilage cells from the epiphysis may be the best cell source for cartilage regeneration [24]. Thus, one-day-old porcine cartilage provided a rich source of cells for research and product development in this study.

The formation of cell sheets by P2 cells at a density of 0.5 × 10^6^ cells/cm^2^ in the supplemented cell culture medium demonstrates the effectiveness of the selected culture conditions. The supplementation with ITS, ascorbate 2-phosphate, dexamethasone, proline, and BSA provided an optimal environment for cell growth and sheet formation. After two weeks of culturing, the decellularization process using a 1% SDS solution effectively removed cellular components, as confirmed by the absence of cellular debris under microscopic examination. The subsequent elimination of genetic material using DNAse/RNAse enzymes ensured the removal of residual DNA and RNA, which is crucial for preventing immune reactions in potential clinical applications.

The freeze-drying process at −80 °C successfully created the PCM biomembrane, which exhibited a uniform structure and retained its mechanical integrity. These characteristics make the PCM biomembrane suitable for further tissue engineering and regenerative medicine applications. The biomembrane’s uniform structure and mechanical integrity are essential for its potential use in cartilage repair and other regenerative therapies. The results indicate that the developed PCM biomembrane holds promise for future clinical applications in tissue engineering and regenerative medicine.

The protein profile analysis revealed that the most recognized protein was associated with ECM1. This finding is significant as ECM1 is essential in preserving tissue structural integrity and promoting cell signaling. Several growth factors were also identified, including TGF, FGF, IGF, and EGF. These growth factors play a role in a range of cellular functions, including cell proliferation, differentiation, and tissue repair. The presence of these proteins and growth factors in the biomembrane suggests its potential efficacy in promoting cartilage repair and regeneration. The results indicate that the PCM biomembrane is a promising candidate for tissue engineering applications, particularly in cartilage repair.

The results of this study demonstrated the effectiveness of different treatments in promoting cartilage repair in the defect area. Cross-imaging and Safranin-O staining revealed the formation of new cartilage tissue in the defect area. No new tissue was observed in the untreated group, indicating that the natural healing process was insufficient for cartilage repair. In the cells-treated group, new tissue with a small number of cells was present, suggesting that introducing chondrocytes alone had a limited effect on cartilage regeneration. No new tissue was observed in the PCM-treated group, indicating that the PCM biomembrane alone was insufficient to induce cartilage repair. However, in the cells/PCM-treated group, new tissue formed in the defect area, with the cells resembling chondrocytes and the lacunae being stained with Safranin-O. This finding suggests that combining chondrocytes and the PCM biomembrane provided a synergistic effect, enhancing cartilage repair and regeneration. In summary, these findings emphasize the promise of utilizing a blend of chondrocytes and the PCM biomembrane to successfully repair cartilage in cases of joint damage.

Biomembranes used in cartilage tissue regeneration for treating autologous chondrocyte implantation (ACI) are crucial in supporting healing and regeneration. They provide a scaffold for chondrocytes (cartilage cells) to adhere to, proliferate, and differentiate, essential for effective cartilage repair. Biomembranes offer a supportive environment for chondrocytes, helping them adhere and grow while protecting them from mechanical and chemical impacts from the surrounding environment. These biomembranes are made from biocompatible materials, such as collagen, chitosan, or other biological polymers, which do not cause immune reactions and are biodegradable.

Using biomembranes enhances the effectiveness of the ACI method by improving chondrocyte adhesion and growth, minimizing the risk of infection and post-surgical complications. Biomembranes have been successfully used in many studies and clinical applications to treat joint cartilage injuries, particularly in the knee. However, there are several limitations associated with the use of biomembranes in cartilage tissue regeneration for ACI: (1) integration issues: biomembranes may not integrate well with the surrounding tissue, leading to poor adhesion and the potential failure of the implant [25,26,27]; (2) immune response: although biomembranes are designed to be biocompatible, there is still a risk of immune response or rejection by the body [28,29]; (3) mechanical properties: the mechanical strength of biomembranes may not match that of natural cartilage, which can lead to issues with durability and functionality [30]; (4) cost and complexity: the production and application of biomembranes can be costly and complex, making them less accessible for widespread clinical use [25,31]; (5) limited long-term data: there are limited long-term data on the effectiveness and safety of biomembranes in ACI, which makes it difficult to assess their potential benefits and risks fully [29,32].

## 5. Conclusions

This study demonstrated a novel approach using cells derived from one-day-old porcine cartilage to fabricate a biomembrane for inducing cartilage repair in knee joints. The cells exhibited high proliferation and renewal, as well as multiple differentiation capabilities, akin to stem cells, which are essential for forming biomembranes. The analysis of the protein profile identified the existence of extracellular structure organization, extracellular matrix organization, collagen fibril organization, and supramolecular fiber organization. Additionally, the PCM biomembrane showed promising results in inducing cartilage repair in joint damage, highlighting its potential as an effective treatment for knee joint injuries.

## Figures and Tables

**Table 1:** Proteomic analysis of PCM biomembrane.

Accession	Gene Name	Description
Q15582	TGFBI	Transforming growth factor-beta-induced protein
Q16610	ECM1	Extracellular matrix protein 1
P35858	IGFALS	Insulin-like growth factor-binding protein complex acid labile subunit
P51858	HDGF	Hepatoma-derived growth factor
Q06210	GFPT1	Glutamine–fructose-6-phosphate aminotransferase [isomerizing] 1
P18065	IGFBP2	Insulin-like growth factor-binding protein 2
Q7Z4V5	HDGFL2	Hepatoma-derived growth factor-related protein 2
Q16270	IGFBP7	Insulin-like growth factor-binding protein 7
Q04756	HGFAC	Hepatocyte growth factor activator
Q13630	GFUS	GDP-L-fucose synthase
Q9P2B2	PTGFRN	Prostaglandin F2 receptor negative regulator
P11717	IGF2R	Cation-independent mannose-6-phosphate receptor
P22692	IGFBP4	Insulin-like growth factor-binding protein 4
P05019	IGF1	Insulin-like growth factor I
Q9Y6M1	IGF2BP2	Insulin-like growth factor 2 mRNA-binding protein 2
P24593	IGFBP5	Insulin-like growth factor-binding protein 5
P61812	TGFB2	Transforming growth factor beta-2 proprotein
P52594	AGFG1	Arf-GAP domain and FG repeat-containing protein 1
P24592	IGFBP6	Insulin-like growth factor-binding protein 6
P49767	VEGFC	Vascular endothelial growth factor C
Q9NZT2	OGFR	Opioid growth factor receptor
P00533	EGFR	Epidermal growth factor receptor
Q9Y3E1	HDGFL3	Hepatoma-derived growth factor-related protein 3
P09619	PDGFRB	Platelet-derived growth factor receptor beta
Q7Z7M0	MEGF8	Multiple epidermal growth factor-like domains protein 8
O00425	IGF2BP3	Insulin-like growth factor 2 mRNA-binding protein 3
P01344	IGF2	Insulin-like growth factor II
Q14512	FGFBP1	Fibroblast growth factor-binding protein 1
Q9NZI8	IGF2BP1	Insulin-like growth factor 2 mRNA-binding protein 1
P56159	GFRA1	GDNF family receptor alpha-1
C9JMX4	IGFBP3	Insulin-like growth factor-binding protein 3 (Fragment)
P16234	PDGFRA	Platelet-derived growth factor receptor alpha
P14136	GFAP	Glial fibrillary acidic protein
A0A0A0MQV6	FGF2	Fibroblast growth factor
P08833	IGFBP1	Insulin-like growth factor-binding protein 1
P01133	EGF	Pro-epidermal growth factor
Q6UW32	IGFL1	Insulin growth factor-like family member 1
Q9GZP0	PDGFD	Platelet-derived growth factor D
Q969H8	MYDGF	Myeloid-derived growth factor
O95081	AGFG2	Arf-GAP domain and FG repeat-containing protein 2
A0A2R8YEI1	HGF	Hepatocyte growth factor
P04085	PDGFA	Platelet-derived growth factor subunit A
Q9NVK5	FGFR1OP2	FGFR1 oncogene partner 2
Q8IUX8	EGFL6	Epidermal growth factor-like protein 6
Q3B7J2	GFOD2	Glucose-fructose oxidoreductase domain-containing protein 2
Q9NRA1	PDGFC	Platelet-derived growth factor C
P49763	PGF	Placenta growth factor
H3BRP2	TGFB1I1	Transforming growth factor beta-1-induced transcript 1 protein
Q63HQ2	EGFLAM	Pikachurin
P55789	GFER	FAD-linked sulfhydryl oxidase ALR

## Data Availability

The original contributions presented in the study are included in the article/Supplementary Materials, further inquiries can be directed to the corresponding author.

## Supplementary Materials

The following supporting information can be downloaded at https://www.mdpi.com/article/10.3390/jfb16030092/s1, Figure S1: The cells were thawed and monitored for morphology under a microscope until the 20th passage (scale bar: 50 µm).

## Abbreviations

The following abbreviations are used in this manuscript:

ACI	autologous chondrocyte implantation
OA	osteoarthritis
ECM	extracellular matrix
GAG	Glycoaminoglycan
PBS	phosphate-buffered saline
P/S	Penicillin–Streptomycin
DMEM	Dulbecco’s Modified Eagle Medium
FBS	fetal bovine serum
ITS	insulin–transferrin–selenium
