# Supplementary material for: Fabrication and Characterization of Immature Porcine Cartilage-Derived Cell Biomembranes
Source: J Funct Biomater. 2025 Mar 5;16(3):92. doi: 10.3390/jfb16030092 (PMC11943366; doi:10.3390/jfb16030092)
Supplement: Supplementary file 1 [file jfb-16-00092-s001.zip › jfb-3471484-supplementary.pdf]

Supplemental materials

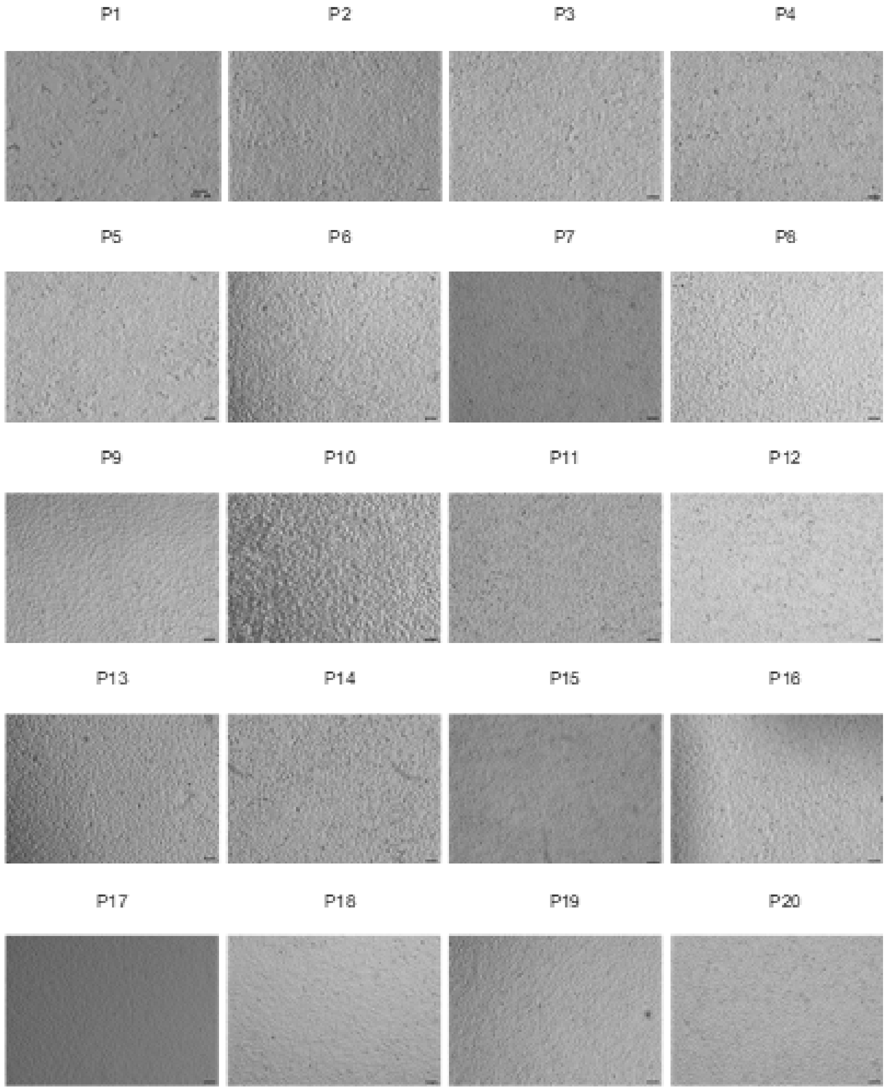

Supplementary Figure S1. The cells were thawed and monitored for morphology under a microscope until the 20th passage (Scale bar: 50 μm).
